# Politics in Public Health: Growing Partisan Divides in COVID-19 Vaccine Attitudes and Uptake Post-2021 Presidential Inauguration

**DOI:** 10.3389/ijph.2025.1608162

**Published:** 2025-05-14

**Authors:** Hongbin Fan, Zhongliang Zhou, Guanping Liu, Chi Shen, Qi Zhang

**Affiliations:** ^1^ School of Public Policy and Administration, Xi’an Jiaotong University, Xi’an, Shaanxi, China; ^2^ School of Community and Environmental Health, Old Dominion University, Norfolk, VA, United States

**Keywords:** political polarization, partisan divides, COVID-19 vaccine attitudes, COVID-19 vaccine uptake, presidential transition

## Abstract

**Objectives:**

To investigate whether the 2021 U.S. presidential inauguration contributed to a widening of partisan divides in COVID-19 vaccine attitudes and uptake.

**Methods:**

We leverage the presidential inauguration as a natural experiment and analyze data from the Household Pulse Survey and CDC vaccination records. Using a difference-in-differences framework with continuous treatment, we examine how the transition differentially affected state-level vaccine refusal rates and county-level vaccination rates, based on varying levels of partisanship as measured by the Trump–Biden vote gap.

**Results:**

Following Biden’s inauguration, vaccine refusal declined more in pro-Biden states. Distrust in government and vaccines accounted for approximately 80% of the interstate variation. County-level analysis revealed that for every 1 percentage point increase in Trump’s vote share over Biden’s, counties experienced an additional 0.515%–2.674% decline in vaccination rates among adults aged 65+. These effects were more pronounced in politically loyal and high-turnout counties.

**Conclusion:**

The presidential transition appears to have widened partisan divides regrading COVID-19 vaccines. These findings highlight the need for depoliticized health messaging and bipartisan strategies to mitigate the influence of partisanship on public health.

## Introduction

Political polarization has been on the rise in the United States. Partisans become increasingly isolated from one another in both lives and ideological thinking. Partisanship is also substantially related to behavior [1, 2]. Nowadays, partisan identity is nearly three times more predictive on a range of social policy issues than other demographic factors [3]. For many Americans, politics has become an extended feature of their social identities, influencing and shaping their behavior in areas seemingly unrelated to politics [4].

With COVID-19 vaccines available to the public by December 2020, vaccination uptake became a public health priority essential to controlling the pandemic. However, the COVID-19 issue in the U.S. was more than just a public health issue [5, 6]. Partisan elites politicized the COVID-19-related issues from the very onset of the pandemic in early 2020 [7], and almost all COVID-19 related attitudes, beliefs, and behaviors have been sharply polarized along party lines [8]. With partisan media often amplifying elite divisions [9], more voters adopted their party’s position on controversial issues [10], and actions like mask-wearing or vaccination became political statements in many communities [11].

While many studies have documented partisan divides in COVID-19 vaccine attitudes [12–16] and uptake [11, 17], the dynamics underlying the formation and intensification of these divides remain underexplored. President Biden’s inauguration presents a unique context to examine how a shift from a Republican to a Democratic administration might catalyze a rapid polarization in vaccine perceptions. This study leverages this political transition as a natural experiment to investigate how elite-driven politicization, amplified by such a high-profile event, can reinforce partisan attitudes toward public health measures in the short term. By focusing on this pivotal period, we aim to provide insights into how executive power transitions impact vaccine attitudes and uptake polarization, contributing to a deeper understanding of how political events shape health-related partisanship.

This study examines partisan shifts in COVID-19 vaccine attitudes and uptake surrounding Biden’s inauguration. We investigate state-level changes in vaccine refusal rates before and after the inauguration in relation to partisan support, and we explore how distrust in government and vaccines may relate to these changes. Additionally, using county-level vaccination data for individuals aged 65 and above, we employ a causal inference analysis to assess the heterogeneous effects of the presidential transition on vaccine uptake in counties with different levels of partisanship.

This research contributes to a deeper understanding of how changes in executive leadership may intensify partisan divides in public health responses, offering insights into the challenges of managing health crises in politically polarized societies.

## Methods

### Data Sources

There are two main data sources in our study. The first main data source is the Household Pulse Survey (HPS), a state-representative survey of individuals. However, it does not include questions about political leanings, so we selected only state-level estimates about COVID-19 vaccine attitudes. New questions regarding COVID-19 vaccine attitude was introduced to the questionnaire beginning with “week” 22 (6–18 January) of the HPS data collection period (biweekly). We thus use data based on the estimates from the 6–18 January and 3–15 February 2021, survey waves to describe a general result about COVID-19 vaccine attitudes.

The second main data source is the CDC data [18], in which we gathered county-level COVID-19 vaccination records and state-level COVID-19 vaccine distribution records from 25 December 2020, to 20 February 2021. Since COVID-19 vaccines were primarily given to healthcare workers and the elderly during this period, and healthcare workers were less representative than the elderly due to their professional knowledge of COVID-19 vaccines, the population aged 65 and older became our study population. With the exception of records from unknown counties, a total of 225,680 vaccination records were initially obtained. We then excluded records with missing values in county-level vote results in the 2020 presidential election and key covariates (e.g., new COVID-19 cases, median household income), as well as a county’s records for that day and the following day if the value for the number of people who received a COVID-19 vaccine in the county on that day was zero or missing. Finally, a total of 68,696 vaccination records from 2,711 counties were included in final analysis.

Based on 5-year estimates from the 2019 American Community Survey by the Census Bureau, we gathered data on county characteristics, including demographic, social, and economic aspects. We gathered county-level social capital characteristics from The Geography of Social Capital (U.S. Congress, 2018). Additionally, we gathered COVID-19 infection and death data from the COVID-19 Impact Analysis Platform at the University of Maryland [19].

### Indicators

We constructed measures of the COVID-19 vaccine refusal rate (CVRR), CVRR due to distrust of the government, and CVRR due to distrust of COVID-19 vaccines using HPS survey data. CVRR was the proportion of people who were “not receiving” or “not planning” to receive a COVID-19 vaccine. CVRR due to distrust of the government and CVRR due to distrust of vaccines were the percentages of residents who would “probably not” or “definitely not” receive a vaccine due to “distrust of the government” and “distrust of COVID-19 vaccines,” respectively.

Using CDC data, we calculated the daily COVID-19 vaccination rate for people aged 65 and above, defined as the daily increase in the percentage of this population receiving at least one vaccine dose.

A continuous index of partisanship was created using the county-level/state-level voting gap in the 2020 election—that is, the proportion of total votes for Donald Trump minus the proportion of total votes for Joe Biden.

Time-varying control variables include new COVID-19 cases per 1,000, active COVID-19 cases per 1,000, COVID-19 death rate and COVID-19 vaccine supply per 100 k (distributed doses minus administrated doses). Time-invariant control variables include demographic characteristics, such as age and race; social characteristics, such as marital status and education attainment; economic characteristics, such as median household income and unemployment rate; and pandemic characteristics, such as 2020 accumulated COVID-19 cases and death cases; and social capital characteristics, such as non-religious non-profit organizations per 1,000 and mail-back census response rates. The descriptive statistics of dependent and main independent variables are described in Table 1, while the descriptive statistics of additional control variables are described in Supplementary Table S1.

**TABLE 1:** Descriptive statistics of dependent and main independent variables (Politics in Public Health, United States, 2020-2021).

Indicators	N	Mean	SD	Median	Min	Max	Level
COVID-19 Vaccine Refusal Rate	100	31.41	5.43	31.26	18.86	44.22	State
COVID-19 Vaccine Refusal Rate due to distrust of the government	100	6.26	1.80	6.31	2.43	10.63	State
COVID-19 Vaccine Refusal Rate due to distrust of COVID-19 vaccines	100	6.63	1.99	6.39	2.10	11.75	State
2020 Trump-Biden Vote Gap	50	0.0232	0.2079	0.0055	−0.3560	0.4366	State
Daily COVID-19 Vaccination Rate	68,696	0.01	0.01	0.01	0.00	0.51	County, Day
COVID-19 Vaccine Supply per 100 k	68,696	5,253.89	1,397.41	5,401.00	0.00	15,268.00	County, Day
New COVID-19 Cases per 1,000	68,696	0.25	0.26	0.19	0.00	23.58	County, Day
Active COVID-19 Cases per 1,000	68,696	14.31	5.66	14.22	0.00	32.68	County, Day
COVID-19 Death Rate	68,696	7.56	2.52	7.06	1.74	16.58	County, Day
2020 Trump-Biden Vote Gap	2,711	0.3009	0.3129	0.3637	−0.8675	0.8893	County
Population	2,711	108,065.10	333,496.80	28,608	668	10100000	County
Proportion Age 65+	2,711	18.80	4.42	18.50	3.20	56.70	County
Proportion Hispanic or Latinx	2,711	6.83	9.30	3.70	0.00	84.20	County
Proportion Black or Africa	2,711	9.41	14.94	2.20	0.00	87.20	County
Proportion Asia	2,711	1.34	2.55	0.60	0.00	39.60	County
Median Household Income	2,711	53,457.38	14,033.10	51,734.00	21,504	142,299	County
Population Density	2,711	233.98	1,344.48	47.00	0.00	48,341.00	County
Urbanization Rate	2,711	0.42	0.31	0.41	0.00	1.00	County

### Empirical Strategy

To demonstrate the growing partisan divide in COVID-19 vaccine attitudes in the month after Biden’s inauguration, we use state-level representative data from HPS to give general results.

To get evidence of growing partisan divide in COVID-19 vaccination, our estimation strategy is logically equivalent to a standard differences-in-differences (DID) strategy. We compared the relative change in daily COVID-19 vaccination rates in the post-Biden period (in the month after Biden’s inauguration) relative to the pre-Biden period between pro-Trump counties and pro-Biden counties. Our estimates differ from a standard DID strategy because we capture more variation in the data by using a continuous measure of the intensity of treatment (the vote gap in the 2020 election). The strategy referring to Qian [20], the regression model is constructed by the DID with continuous treatment in this study as shown in Equation 1:

$$\log\left({DCVR}_{it}\right)=\beta_{0}+{\beta_{1}\cdot VG}_{i}\cdot I_{t}^{post}+X_{it}\cdot \tau +\sum_{j=-26}^{31}X_{i}\cdot I_{t}^{j}\cdot \Phi_{j}+\sum_{c}\delta_{c}\cdot I_{i}^{c}+\sum_{j=-26}^{31}\gamma_{t}\cdot I_{t}^{j}+\varepsilon_{it}$$ (1)

Where:

• $\log\;\left({DCVR}_{it}\right)$ denotes the logarithmic form of daily COVID-19 vaccination rate for each county *i* admitted at day *t.* We take the natural log of the variables to remove the skewness that exists in their distributions otherwise.
• ${VG}_{i}$ denotes the vote gap in the 2020 election for each county *i.*
• $I_{t}^{post}$ is a dummy variable equaling 1 for the periods after 20 January 2021, otherwise is 0.
• $\sum_{j=-26}^{31}\gamma_{t}\cdot I_{t}^{j}$ and $\sum_{c}\delta_{c}\cdot I_{i}^{c}$ are time and county fixed effects.
• $X_{it}$ are a series of time-varying control variables including vaccine supply, daily new COVID cases, COVID death rate, active COVID cases.
• $\sum_{j=-26}^{31}X_{i}\cdot I_{t}^{j}\cdot \Phi_{j}$ are county-specific characteristics (time-invariant control variables) interacted with time-period fixed effects
• Estimation is generally performed with standard errors clustered at the county level and weighted by population.

The coefficient of interest in Equation 1 is $\beta_{1}$ , which measures the additional growth in daily COVID-19 vaccination rate experienced by counties that voted more for Trump (relative to those that voted more for Biden) in the month after Biden’s inauguration (relative to before). A negative coefficient indicates that counties that voted more for Trump witnessed an additional less COVID-19 vaccination in the month after Biden’s inauguration, relative to before.

Equation 1 examines the average effect of partisanship on vaccination in the month after Biden’s inauguration. Referring to Nunn [21], our strategy for providing a precise estimate of the dynamic treatment effect and testing the hypothesis of parallel trend is to estimate a fully flexible estimating equation that takes the following form:

$$\log\left({DCVR}_{it}\right)=\beta_{0}+\sum_{j=-26}^{31}\beta_{j}\cdot {VG}_{i}\cdot I_{t}^{j}+X_{it}\cdot \tau +\sum_{j=-26}^{31}X_{i}\cdot I_{t}^{j}\cdot \Phi_{j}+\sum_{c}\delta_{c}\cdot I_{i}^{c}+\sum_{j=-26}^{31}\gamma_{t}\cdot I_{t}^{j}+\varepsilon_{it}$$ (2)

where all variables are defined as in Equation 1. The only difference from Equation 1 is that in Equation 2, rather than interacting ${VG}_{i}$ with a post-Biden indicator variable, we interact vote gap measure with each of the time-period fixed effects. The estimated vectors of $\beta_{j}s$ reveal the correlation between vote gap and the outcomes of interest for each time period. If partisan difference in daily COVID-19 vaccination rate was aggravated in the month after Biden’s inauguration, then we would expect the estimated $\beta_{j}s$ to be constant over time for the days before Biden’s inauguration and to be larger in magnitude for the days after the inauguration.

## Results

### Growing Partisan Divide in COVID-19 Vaccine Attitudes

**FIGURE 1:**
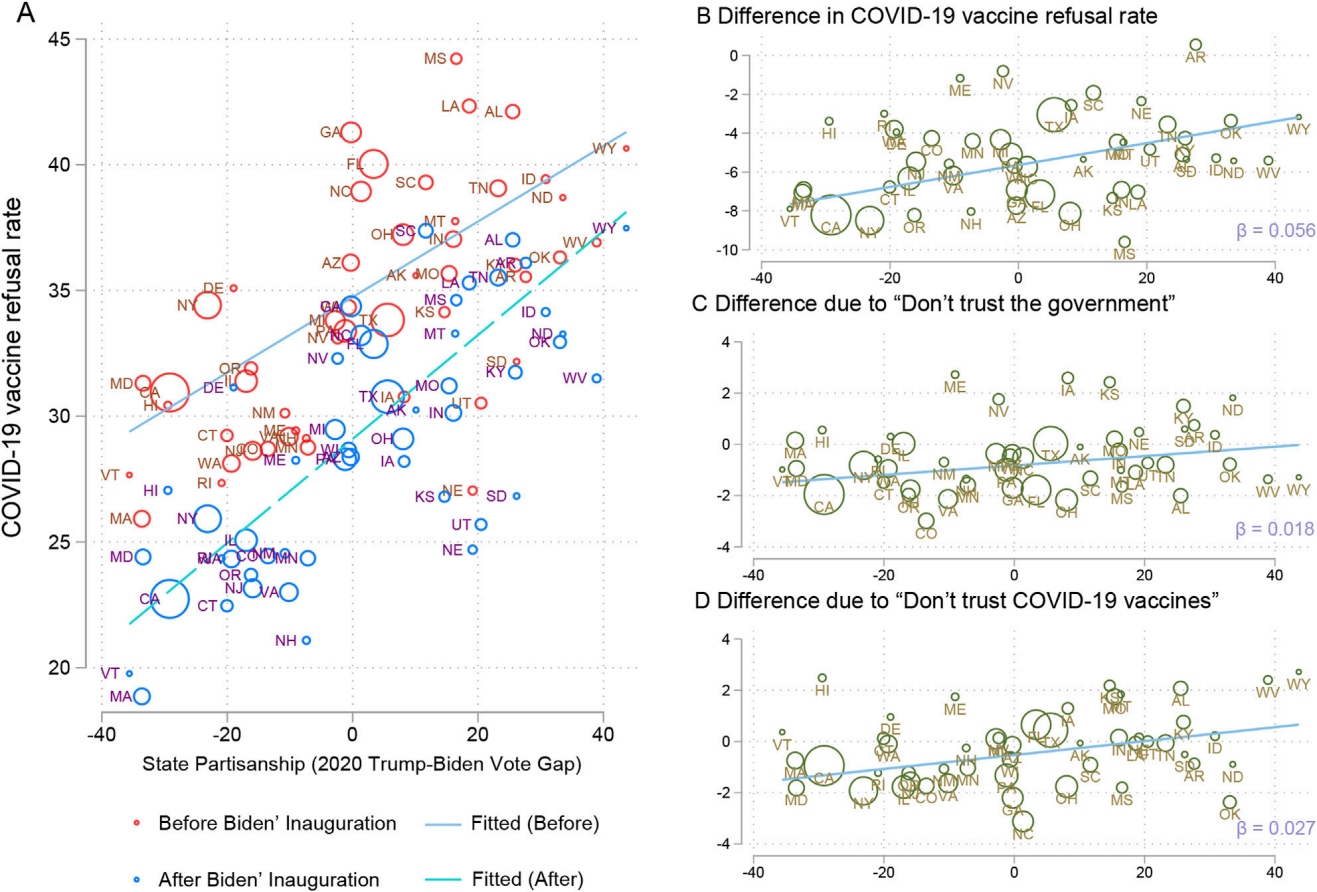
US states’ COVID-19 vaccine attitudes and the COVID-19 vaccine attitude gaps before and after the presidential inauguration as a function of partisanship (Politics in Public health, United States, 2020-2021). COVID-19 vaccine attitudes were quantified as states’ COVID-19 vaccine refusal rates before and after Biden’ inauguration **(A)**. The COVID-19 vaccine attitude gaps before and after Biden assumed office were quantified as the states’ differences in COVID-19 vaccine refusal rates before and after Biden’ inauguration **(B)**, the differences in COVID-19 vaccine refusal rates due to “Don’t trust the government” before and after Biden’ inauguration **(C)**, and the differences in COVID-19 vaccine refusal rates due to “Don’t trust COVID-19 vaccines” before and after Biden’ inauguration **(D)**. Partisanship was quantified as the states’ voting gap in the 2020 election. Each circle represents a state, its size proportional to its population.

Using data from the HPS survey, Figure 1A shows that both before and after Biden took office, COVID-19 vaccine refusal rates were consistently higher in states that voted more for Trump in the 2020 election compared to states that voted more for Biden. However, in the month after Biden’s inauguration, the correlation between COVID-19 vaccine refusal rates and vote gaps grew stronger (the coefficient increased from 0.15 to 0.21).

To better demonstrate the heterogeneous effects of Biden’s taking office on COVID-19 vaccine attitudes in states with different levels of partisanship, we compared the differences in COVID-19 vaccine refusal rates before and after Biden took office between states. The results in Figure 1B show that COVID-19 vaccine refusal rates declined in almost every state in the month after Biden’s inauguration, but the magnitude of the decline varied greatly: states that voted more for Trump showed obviously fewer declines (e.g., California showed an over eight absolute percentage points decline, while Texas about three). Specifically, for every 1 percentage point increase in vote share for Donald Trump over Joe Biden, the drop in COVID-19 vaccine refusal rates would be reduced by 0.056 absolute percentage points on average (about 370 k people).

In addition, we compared the differences in COVID-19 vaccine refusal rates due to “Don’t trust the government” and “Don’t trust COVID-19 vaccines” before and after Biden’s inauguration between states. The result in Figures 1C,D shows that, similar to the results above, states that voted more for Trump had lower drops in vaccine refusals for both reasons in the month after Biden’s inauguration. Specifically, the drop in vaccine refusal rates due to “Don’t trust the government” and “Don’t trust COVID-19 vaccines” would be lowered by 0.018 absolute and 0.027 absolute percentage points on average for every 1 percentage point increase in vote share for Donald Trump over Joe Biden, respectively.

### Growing Partisan Divide in COVID-19 Vaccine Uptake

The average impact of Biden’s taking office on partisan differences in daily COVID-19 vaccination rates among older adults (65+) can be inferred from the DID results in Table 2. The first specification, reported in column [1], consists of time-period fixed effects, county and state fixed effects, clusters in counties, and no controls. In column [2], we report estimates for our baseline specification, including main control variables, which are made up of time-varying control variables and time-invariant demographic and socioeconomic control variables (interacted with the time-period fixed effects). The coefficient is −0.879, which implies that in the month after Biden’s inauguration, for every 1 percentage point increase in vote share for Donald Trump over Joe Biden, counties exhibited an additional 0.879 percent less COVID-19 vaccination among older adults (65+). Column [3, 4] report the robustness of our results to clustering in state and the introduction of additional control variables. We further analyze the data by splitting it into two datasets based on governor partisanship and analyzing each of them separately in order to rule out bias resulting from variations in vaccine availability. Detailed results can be found in the Supplementary Material.

**TABLE 2:** The impact of presidential change and Biden’s actions: Baseline estimates (Politics in Public Health, United States, 2020-2021).

Ln daily COVID-19 vaccination rate				
	(1)	(2)	(3)	(4)
Voting gap × Post	−0.572^***^ (0.1668)	−0.879^***^ (0.168)	−0.879^***^ (0.2648)	−0.871^***^ (0.2796)
Controls				
Supply	No	Yes	Yes	Yes
New cases	No	Yes	Yes	Yes
Active cases	No	Yes	Yes	Yes
Death rate	No	Yes	Yes	Yes
Baseline controls (× day fixed effects)				
Ln (population)	No	Yes	Yes	Yes
Age	No	Yes	Yes	Yes
Race	No	Yes	Yes	Yes
Income	No	Yes	Yes	Yes
Population density	No	Yes	Yes	Yes
Urbanization rate	No	Yes	Yes	Yes
Additional controls (× day fixed effects)				
	No	No	No	Yes
Fixed effect	Yes	Yes	Yes	Yes
cluster	County	County	State	County
Observations	2,672	2,601	2,601	2,601
N	68,657	61,228	61,228	60,050
R-squared	0.394	0.464	0.464	0.567
Adjusted R-squared	0.369	0.435	0.435	0.528

**FIGURE 2:**
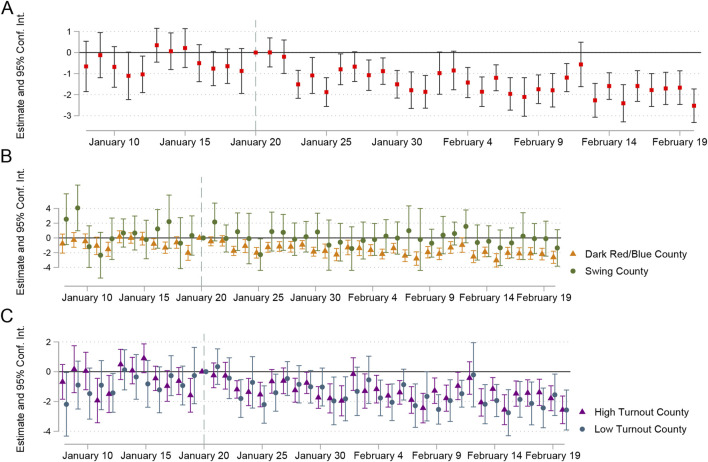
Main analysis and heterogeneity analysis (Politics in Public Health, United States, 2020-2021). **(A)**: Flexible estimates and 95% Conf. Inf. of the partisan difference in vaccination behaviors (daily COVID-19 vaccination rates) between 8 January and 20 February 2021. Small red squares are the estimates and black solid lines are 95% Conf. Inf. of point estimates, daily COVID-19 vaccination rates are log-transformed. **(B)**: Flexible estimates and 95% Conf. Inf. of the partisan difference in vaccination behaviors (daily COVID-19 vaccination rates) in dark red/blue counties and swing counties between 8 January and 20 February 2021. **(C)**: Flexible estimates and 95% Conf. Inf. of the partisan difference in vaccination behaviors (daily COVID-19 vaccination rates) in high turnout counties and low turnout counties between 8 January and 20 February 2021.

From flexible estimates, a clear pattern emerges from Figure 2A. The relationship between vote gap and daily COVID-19 vaccination rates among older adults was stable before Biden’s inauguration. The magnitude of the estimate then sharply increased on January 23 (COVID Data Tracker’s vaccination data typically has a lag time [22]), keeping it dynamically stable at another level. Specifically, in the month after Biden’s inauguration, for every 1 percentage point increase in vote share for Donald Trump over Joe Biden, counties exhibited an additional 0.515%–2.674% less COVID-19 vaccination among older adults. We learn two important facts from the fully flexible estimates. First, we do not observe any clear trends in the estimated interaction effects during the time periods immediately prior to Biden’s inauguration. The second insight we gain is that the gap between counties that voted more for Trump and those that voted more for Biden in daily COVID-19 vaccination rates among older adults widened immediately after Biden took office.

Furthermore, we conducted additional analysis to address two other questions that interested us: whether counties with higher political loyalty and whether counties with higher political participation were more adversely affected, and therefore showed more exacerbated partisan differences in COVID-19 vaccine uptake in the month after Biden’s inauguration.

We call those counties that have consistently voted for one party since 2000 dark red/blue counties, and the others swing counties. Figure 2B shows that the exacerbated difference in daily COVID-19 vaccination rates between dark red and dark blue counties was noticeably greater than between swing counties, and the latter was almost statistically insignificant. Similarly, we refer to the counties with a turnout rate of over 50% as “high turnout counties” and the remainder as “low turnout counties.” Results in Figure 2C demonstrate that the partisan differences in daily COVID-19 vaccination rates were also exacerbated more severely in high turnout counties.

## Discussion

This study suggests that partisan divides in COVID-19 vaccine attitudes and uptake may have grown wider in the month following Biden’s inauguration. Specifically, a greater proportion of individuals in pro-Biden states, compared to pro-Trump states, shifted from vaccine refusal to acceptance, with around 80% of inter-state variation explained by distrust of government and COVID-19 vaccines, both likely catalyzed by Biden’s presidency and his administration’s policy actions. Moreover, as the partisan divide in vaccine attitudes appeared to intensify, the divide in vaccine uptake may have widened as well. Our analysis revealed that for every percentage point increase in Trump’s vote share over Biden’s, counties saw an additional 0.515%–2.674% reduction in COVID-19 vaccination among older adults, with the impact most pronounced in counties characterized by high political loyalty.

**FIGURE 3:**
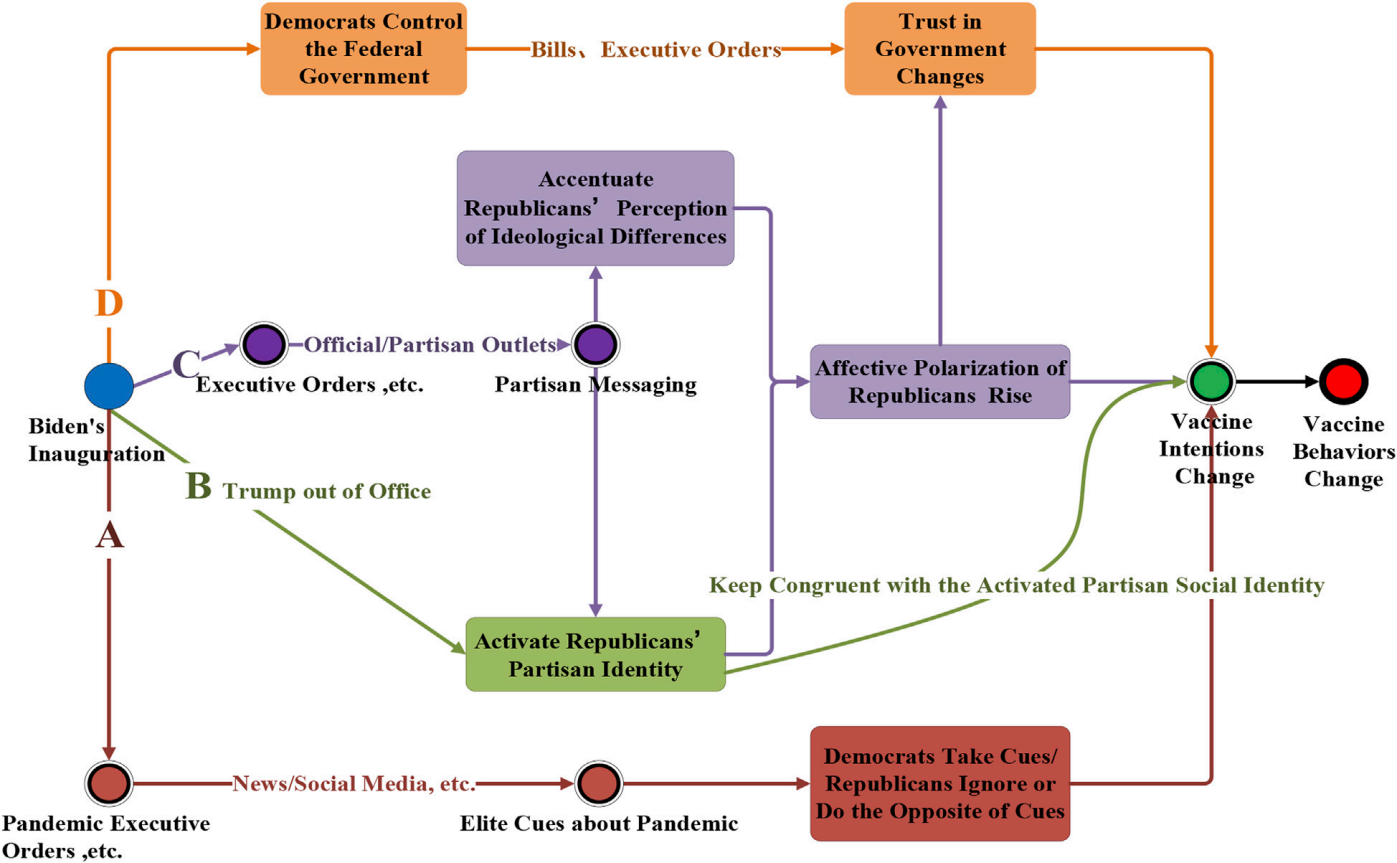
Potential theoretical pathways through which the presidential change and the actions of the Biden administration could exacerbate partisan differences in intention and behavior to vaccinate against COVID-19 (Politics in Public Health, United States, 2020-2021).

The potential exacerbation of partisan differences may stem from several theoretical pathways, as illustrated in Figure 3. First, under elite cues theory, Biden’s initial executive actions—such as the “100 Days Masking Challenge” and initiatives to increase vaccination supply—could have effectively promoted vaccination among Democrats while having limited influence on Republicans, potentially even backfiring and intensifying the partisan divide [16]. Counties with higher levels of political loyalty might have been more affected due to greater sensitivity to elite cues [23].

Social identity theory offers another possible explanation. Partisan social identities are often heightened during times of political transition [24]. Biden’s inauguration might have reinforced Republicans’ partisan identity, potentially leading them to resist vaccination efforts associated with Democratic initiatives [25]. Conversely, Democrats may have interpreted the political shift as an alignment with their values, reinforcing support for vaccination.

**FIGURE 4:**
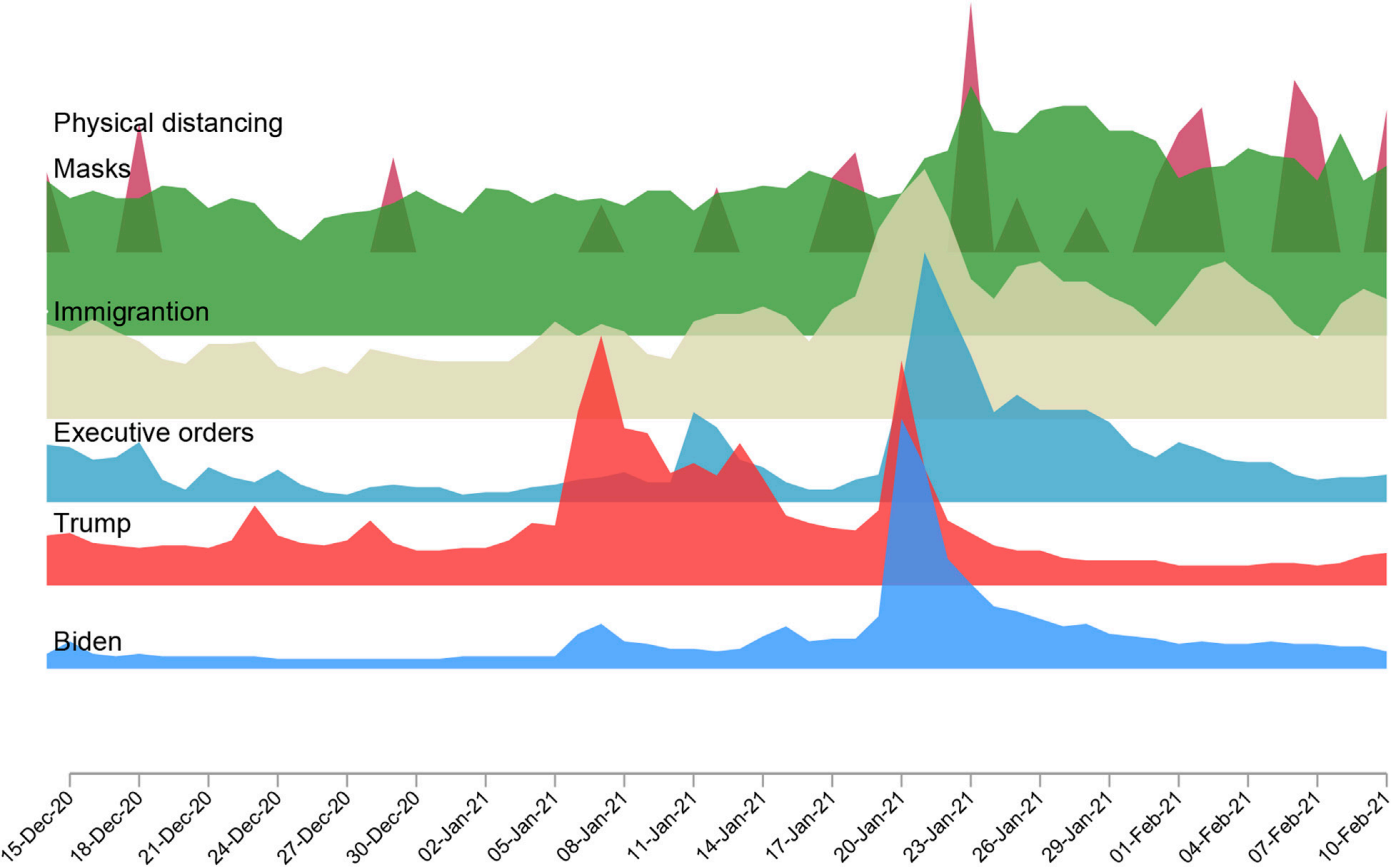
Google searches for hot individuals and hot words before and after Biden’ inauguration from 14 December 2020, to 10 February 2021 (Politics in Public Health, United States, 2020‐2021).

Affective polarization, or the intensifying animosity between partisan groups, could also have played a significant role. Political events such as elections and inaugurations tend to amplify affective polarization by increasing political conflict and media discourse [3, 26]. Figure 4 shows the dramatic increase in the relative number of Google searches on these people and issues. As Biden assumed office, heightened hostility toward Democrats among Republicans may have further motivated vaccine hesitancy among Republicans, deepening the partisan divide in public health compliance [27].

Finally, partisan identification may have affected vaccine willingness through varying levels of trust in government [16, 28]. As Biden took office, Democrats’ trust in government could have increased, while Republicans’ trust declined, leading to a broader partisan gap in vaccine uptake.

There could be other reasons for the potential exacerbated partisan differences. However, we argue that the impact of these reasons may be weaker. First, some individuals had already received COVID-19 vaccinations prior to Biden’s inauguration. Compared to Republicans, vaccinated Democrats were more likely to encourage others to get vaccinated [16], while Republicans were more likely to perceive and report adverse events associated with the vaccine [6]. These differences may have contributed to a greater decline in vaccine refusal rates in pro-Biden states. However, at the time, COVID-19 vaccination coverage was still too low [29] to explain the substantial partisan differences observed. Furthermore, the recommendation effect may plausibly account for partisan divide in declining COVID-19 vaccine refusal rates due to distrust of COVID-19 vaccines, but not for partisan divide in declining COVID-19 vaccine refusal rates due to distrust of the government. Second, the supply of COVID-19 vaccines also increased in the month after Biden’s inauguration, which might result in daily COVID-19 vaccination rates exhibiting more of the previously inherent partisan differences in COVID-19 vaccine attitudes. However, if partisan differences in COVID-19 vaccine attitudes had not worsened, we would expect a steady widening trend in daily COVID-19 vaccination rates, not the sharp increase in partisan differences observed in our results. Additionally, the supply of COVID-19 vaccines at the state level was also controlled in our model.

The heavy politicization of COVID-19 vaccines remains a core reason for these divides [11]. Although Biden called for nonpartisan support for vaccination, his Democratic identity may limit his influence on Republican attitudes [30–32]. Depoliticizing public health issues, therefore, becomes essential to foster trust across party lines. For instance, bipartisan messaging, as seen in former President Trump’s March 2021 call for vaccination, can be a promising example [33, 34]. However, remedying the impacts of vaccine politicization requires consistent pro-vaccine messaging from Trump and other Republican elites. Such cues have been shown to effectively increase vaccination rates among Republicans [35], though Republican elite engagement in this effort remains limited.

Although the public had long known that Joe Biden would be inaugurated in January 2021, the event may still have contributed to a widening of partisan divides in COVID-19 vaccine attitudes and uptake. This suggests that affective partisan responses to political events, such as leadership transitions, also drive intensifying partisan divides. While affective responses to political events are a natural byproduct of democratic politics and not necessarily bad [26], it does not mean their negative impact cannot be mitigated. Reducing partisan framing and biases rooted in in-group versus out-group thinking is essential for managing these responses [36]. Approaches that stress shared identities, such as American identity [37], or counter misperceptions about partisan groups [38] could help depolarize public health responses. This implies that during political events like presidential transitions, public health messaging should aim to bridge partisan divides, emphasizing common health goals and avoiding politicized framing.

This study’s focus on the short-term impact of a presidential transition offers insight into how political polarization influences public health policy. Future health communication strategies should consider the broader political context and proactively work to reduce the influence of partisan identities on health behaviors. Long-term solutions may include bipartisan health campaigns and cross-party engagement in public health messaging to create unified responses to crises.

One limitation of this study is that the county/state-level analysis is ecological in nature, so the findings should be interpreted with caution. Additionally, due to data limitations, sensitivity analyses for the vaccine attitude outcomes could not be conducted. Future research with more granular data is necessary to address this issue. Moreover, we used COVID-19 vaccination rates among individuals aged 65 and older as a proxy for overall vaccine uptake, as vaccination for the general population had not yet begun at the time of the presidential transition. Seniors have distinct patterns of partisan identity, with higher levels of partisanship [39] and stronger partisan loyalty due to the life-cycle effect [40] and longer attachments to political parties [41]. Moreover, seniors' health insurance status and media consumption habits differ from those of other age groups. As such, our findings on vaccination uptake may not be fully representative of the general population. Furthermore, the mechanisms underlying these patterns require further investigation. Future studies should aim to verify the relative impact of these mechanisms by analyzing pre- and post-presidential change surveys, incorporating measures of affective polarization, trust in government, and other relevant variables.

### Conclusion

This study suggests that, following President Biden’s inauguration, partisan divides in COVID-19 vaccine attitudes and uptake among older adults (65+) may have widened, potentially driven by elite cues, partisan identity, affective polarization, and trust in government. These findings highlight the importance of depoliticized public health messaging, bipartisan leadership, and targeted interventions in politically polarized contexts. Practical implications include the need to rebuild trust in government, foster cross-party collaboration, and reframe public health initiatives around shared national goals.

Given the study’s focus on older adults, future research should examine vaccine attitudes and uptake among younger populations, who may be shaped by different political socialization processes. Such studies could explore additional political variables, including ideological extremity, susceptibility to misinformation, media consumption habits, and the influence of social media on political attitudes. This would offer a more comprehensive understanding of how political identities shape health behaviors across different demographic groups.
