# Supplementary material for: Politics in Public Health: Growing Partisan Divides in COVID-19 Vaccine Attitudes and Uptake Post-2021 Presidential Inauguration
Source: Int J Public Health. 2025 May 14;70:1608162. doi: 10.3389/ijph.2025.1608162 (PMC12116312; doi:10.3389/ijph.2025.1608162)
Supplement: Supplementary file 1 [file DataSheet1.docx]

**Table. S1 Descriptive statistics of additional control variables**

| Indicators | N | Mean | SD | Median | Min | Max | Level |
| --- | --- | --- | --- | --- | --- | --- | --- |
| Proportion Age 0-17 | 2711 | 22.10 | 3.25 | 22.10 | 7.30 | 41.80 | County |
| Median Age | 2711 | 41.53 | 5.14 | 41.40 | 23.40 | 67.40 | County |

| Proportion Married-couple Household | 2711 | 50.39 | 6.58 | 50.80 | 21.30 | 80.40 | County |
| --- | --- | --- | --- | --- | --- | --- | --- |
| Proportion Households with one or more people under 18 | 2711 | 29.13 | 5.22 | 29.00 | 6.30 | 54.40 | County |
| Proportion Households with one or more people over 65 | 2711 | 33.13 | 5.77 | 32.90 | 12.10 | 77.20 | County |
| Proportion Males 15+ Married | 2711 | 52.11 | 7.03 | 52.90 | 22.20 | 75.50 | County |
| Proportion Females 15+ Married | 2711 | 50.55 | 7.20 | 51.20 | 18.10 | 75.30 | County |
| Proportion of population 25+ with more than a high school education | 2711 | 87.37 | 5.64 | 88.50 | 56.90 | 98.10 | County |
| Proportion of population 25+ with more than a bachelor education | 2711 | 22.10 | 9.54 | 19.60 | 5.40 | 75.30 | County |
| Disability Rate | 2711 | 16.02 | 4.38 | 15.50 | 5.00 | 36.80 | County |
| Unemployment Rate | 2711 | 5.29 | 2.50 | 5.00 | 0.00 | 24.90 | County |
| Percent of Management, Business, Science, and Arts occupations | 2711 | 32.36 | 6.58 | 31.40 | 16.10 | 70.10 | County |
| Percent of Service occupations | 2711 | 17.89 | 3.35 | 17.50 | 6.60 | 41.00 | County |
| Percent of Sales and Office occupations | 2711 | 20.23 | 2.70 | 20.30 | 9.00 | 33.80 | County |
| Percent of Natural resources, Construction, and Maintenance occupations | 2711 | 12.17 | 3.77 | 11.90 | 2.20 | 33.40 | County |
| Percent of Production, Transportation, and Material moving occupations | 2711 | 17.34 | 5.86 | 17.00 | 3.00 | 40.40 | County |
| Percent of Employees in Agriculture & Forestry & Fishing & Hunting & mining | 2711 | 5.70 | 6.17 | 3.60 | 0.00 | 46.40 | County |
| Percent of Employees in Construction | 2711 | 7.32 | 2.19 | 7.10 | 0.40 | 20.30 | County |
| Percent of Employees in Manufacturing | 2711 | 13.06 | 7.10 | 12.20 | 0.00 | 47.50 | County |
| Percent of Employees in Wholesale Trade | 2711 | 2.40 | 1.19 | 2.30 | 0.00 | 19.10 | County |
| Percent of Employees in Retail Trade | 2711 | 11.30 | 2.24 | 11.30 | 1.40 | 25.70 | County |
| Percent of Employees in Transportation and Warehousing, and Utilities | 2711 | 5.55 | 1.91 | 5.30 | 0.00 | 18.90 | County |
| Percent of Employees in Information | 2711 | 1.36 | 0.77 | 1.30 | 0.00 | 11.60 | County |
| Percent of Employees in Finance & Insurance & Real Estate & Rental & Leasing | 2711 | 4.59 | 1.90 | 4.30 | 0.00 | 20.10 | County |
| Percent of Employees in Professional & Scientific & Management & Administrative & Waste Management Services | 2711 | 7.07 | 3.20 | 6.50 | 0.40 | 30.00 | County |
| Percent of Employees in Educational Services, Health Care and Social Assistance | 2711 | 23.38 | 4.55 | 23.00 | 8.10 | 57.90 | County |
| Percent of Employees in Arts & Entertainment & Recreation & Accommodation & Food Services | 2711 | 8.28 | 3.34 | 8.00 | 0.00 | 33.60 | County |
| Percent of Employees in Public Administration | 2711 | 5.39 | 2.93 | 4.70 | 0.80 | 33.10 | County |
| Proportion of Private wage and salary workers | 2711 | 76.04 | 6.93 | 77.20 | 26.80 | 88.70 | County |
| Proportion of Government workers | 2711 | 16.33 | 5.75 | 15.20 | 5.60 | 65.40 | County |
| Proportion of Self-employed in own not incorporated business workers | 2711 | 7.36 | 3.46 | 6.50 | 1.60 | 33.90 | County |
| Proportion of Unpaid Family workers | 2711 | 0.27 | 0.37 | 0.20 | 0.00 | 6.50 | County |
| Mean Household Income | 2711 | 70036.65 | 17338.36 | 66718.00 | 35819.00 | 176019.00 | County |
| Poverty Rate | 2711 | 15.11 | 6.27 | 14.20 | 2.80 | 55.50 | County |
| 2020 Accumulated COVID-19 Cases | 2711 | 0.08 | 0.03 | 0.08 | 0.00 | 0.46 | County |
| 2020 Accumulated COVID-19 Death Cases | 2711 | 0.01 | 0.00 | 0.01 | 0.00 | 0.03 | County |

| Non-religious Non-profit Organizations per 1000 | 2711 | 4.76 | 2.68 | 4.10 | 0.30 | 28.00 | County |
| --- | --- | --- | --- | --- | --- | --- | --- |
| Religious Congregations per 1000 | 2711 | 2.32 | 1.28 | 2.10 | 0.40 | 10.60 | County |
| Mail-back Census Response Rates | 2690 | 71.79 | 10.23 | 75.00 | 22.00 | 95.00 | County |
| Violent Crimes per 100k | 2625 | 245.08 | 192.42 | 196.10 | 9.02 | 1717.70 | County |

To eliminate bias arising from differences in vaccine availability, we further analyze the data by dividing it into two datasets based on governor partisan and analyzing each of them separately. The results prove that this phenomenon of widening partisan differences in vaccination exists in states with different governor partisanship, although not to the same extent (as shown in Table. S2 and Figure.S1). The estimates for our baseline specification are shown in Column (1); the estimates with the governor partisanship control variable added are shown in Column (2); and the estimates of the sub-dataset are shown in Columns (3) and (4), using the datasets with Democratic and Republican governors, respectively.

The results show that the widening of partisan differences in vaccinations is more pronounced in those counties with Republican governor than in counties with Democratic governor, which is logically consistent with our mechanism analysis that counties where the governor is Republican may pick up more elite cues and partisan messages.

**Table. S2 The impact of presidential change and Biden’s actions: Sub-dataset Estimates**

| Ln daily COVID-19 vaccination rate | | | | |
| --- | --- | --- | --- | --- |
|  | (1) | (2) | (3) | (4) |
| Voting gap × Post | -0.879^***^  （0.168） | -0.879^***^  （0.18） | -0.613^***^  （0.21） | -1.047^***^  （0.3646） |
| Controls | | | | |
| Supply | Yes | Yes | Yes | Yes |
| New cases | Yes | Yes | Yes | Yes |
| Active cases | Yes | Yes | Yes | Yes |
| Death rate | Yes | Yes | Yes | Yes |
| Baseline controls (× day fixed effects) | | | | |
| Ln (population) | Yes | Yes | Yes | Yes |
| Age | Yes | Yes | Yes | Yes |
| Race | Yes | Yes | Yes | Yes |
| Income | Yes | Yes | Yes | Yes |
| Population density | Yes | Yes | Yes | Yes |
| Urbanization rate | Yes | Yes | Yes | Yes |
| Additional controls (× day fixed effects) | | | | |
|  | No | No | No | No |
| Fixed effect | Yes | Yes | Yes | Yes |
| cluster | County | County | County | County |
| Observations | 2601 | 2600 | 1173 | 1427 |
| N | 61288 | 61244 | 31300 | 29943 |
| R-squared | 46.38 | 48.10 | 58.23 | 42.00 |
| Adjusted R-squared | 43.48 | 45.25 | 55.88 | 38.01 |


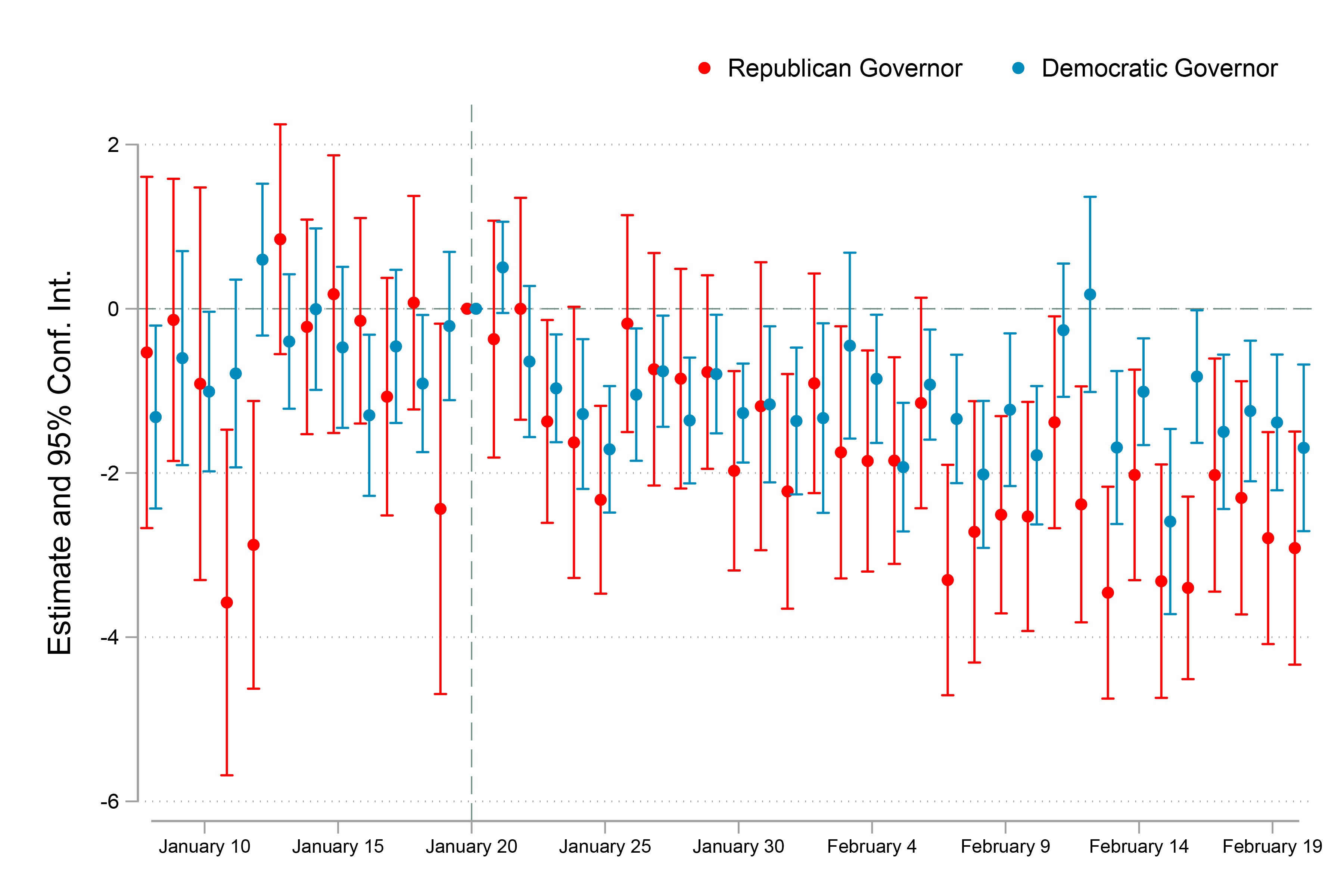


**Figure. S1 Sub-dataset flexible DID estimates**
